# Postural imbalance without visual input is associated with specific neuropsychological deficits in older adults – results from the LIFE-adult study

**DOI:** 10.3389/fneur.2024.1452150

**Published:** 2024-12-11

**Authors:** Eva Grill, Andreas Zwergal, Dorothee Saur, Julian Klingbeil, Christopher Fricke, Florian Schöberl, Karim Felfela, Andrea Zülke, Steffi Riedel-Heller, Joseph Classen

**Affiliations:** ^1^Institute for Medical Information Processing, Biometry, and Epidemiology, Faculty of Medicine, Ludwig-Maximilians Universität Munich, Munich, Germany; ^2^German Center for Vertigo and Balance Disorders, LMU University Hospital, Munich, Germany; ^3^Department of Neurology, LMU University Hospital, Munich, Germany; ^4^Department of Neurology, Leipzig University Medical Center, Leipzig, Germany; ^5^Institute for Social Medicine, Occupational Health and Public Health, Medical Faculty, University of Leipzig, Leipzig, Germany

**Keywords:** Hans Straka’s memory, the vestibular system, mild cognitive impairment, cognition, postural balance

## Abstract

**Introduction:**

Modifiable risk factors play an important role in preventing dementia and reducing its progression. Regular physical activity already in midlife, which relies on intact multisensory balance control, can help to decrease the risk of dementia. However, our understanding of the relationship between postural balance and cognitive functions remains limited. The objective of our study was to investigate the association of postural balance during different sensory conditions with specific cognitive domains in older adults.

**Methods:**

Participants were from the population-based prospective “Leipzig Research Center for Civilization Diseases” (LIFE-Adult) cohort in Leipzig, Germany. Executive, memory and processing speed functions were tested by the Consortium to Establish a Registry for Alzheimer’s disease (CERAD) plus battery. Assessment of visuospatial abilities was based on the short form of the Judgment of Line Orientation Test (JLO). Postural sway was recorded on a force plate with eyes open and closed. Romberg’s ratios were calculated for sway path and sway area as a proxy for balance without visual control and tested in generalized linear regression models with the summary scores of executive function, memory, processing speed and visuospatial function as dependent variables. All models were adjusted for sex, age, ApoE status, socioeconomic status, anamnestic stroke, and diabetes.

**Results:**

In total, we analyzed 460 participants with a mean age of 68.6 years, range 60 to 80, 47.6% female. A higher Romberg’s ratio for sway area was a significant indicator for impaired visuospatial abilities as measured by the dichotomized JLO (Odds Ratio = 1.42, 95% confidence interval 1.07 to 1.88). Romberg’s ratios were not significantly associated with executive functions, procedural speed or memory functions.

**Discussion:**

It may be worthwhile to examine in the future whether inclusion of balance testing enhances the value of screening programs for cognitive impairment. Inversely, it may be appropriate to apply routine cognitive tests when balance problems are detected in older patients.

## 1 Introduction

Dementia is among the major causes for disability and need for care worldwide (1). Over 55 million individuals currently have dementia, with an expected increase of 10 million new cases per year (2). The World Health Organization reported that the costs of caring for the estimated 14.1 million people living with dementia in Europe amounted to US$ 439 billion in 2019 or US$ 32,144 per individual (3), which is close to the mean Gross Domestic Product *per capita* in Europe [US$ 34,160 in 2022].

Physical inactivity, hypertension, obesity, midlife diabetes, hyperlipidemia, and hearing loss have been identified as major modifiable risk factors for preventing dementia and reducing its progression (4, 5). A healthy lifestyle already in midlife, which critically includes regular physical activity, can help to decrease the risk of dementia (5).

Being physically active relies on an intact balance, which is necessary for upright posture, steady bipedal walking, and a stable representation of the environment. To keep the body in balance, output from visual, proprioceptive, and vestibular systems gets integrated. Decline in vestibular or proprioceptive function can affect posture and gait patterns, increase the risk of falling and as such affect daily functioning, participation and quality of life (6).

The interaction of the multisensory and cognitive function may be at least two-fold. Sensory disorders may affect cognition directly via shared cerebral networks or indirectly via alteration of physical activity. In the last decades, for example, it has been demonstrated that deficits of vestibular function impact several cognitive domains including spatial navigation (7–9), short-term memory and executive function (10), and attention and visuospatial abilities (11). Recent studies linked decreased postural balance (12) and impaired vestibular function (13) to dementia. If this association holds for both postural balance and multisensory vestibular function, early disturbances of sensory balance control need to be identified already in middle-aged and older adults to allow preventive interventions. There is a growing body of evidence that both dedicated balance rehabilitation programs (14) and interventions focusing on vestibular perceptual training improve postural balance (15, 16) possibly also mitigating the risk of dementia. Furthermore, non-invasive vestibular or proprioceptive stimulation devices can improve postural balance (17, 18).

Despite these traces of evidence, our understanding of the relationship between postural balance and cognitive functions remains limited. Little is known about whether certain cognitive domains are disproportionally affected in individuals with impaired postural balance. This question is relevant, because identifying associations with specific cognitive domains could impact diagnostic and therapeutic strategies for patients with combined balance and cognitive disorders. Therefore, the objective of our study was to investigate the association of postural balance during different sensory conditions with specific cognitive domains in older adults.

## 2 Methods

### 2.1 Study design and participants

Participants were from the population-based prospective “Leipzig Research Center for Civilization Diseases” (LIFE-Adult) Study in Leipzig, Germany (19). Adult inhabitants of the city of Leipzig were randomly invited via the population registry. Participants underwent structured neuropsychological testing and medical examinations. Medical history and medication were assessed by structured interview. A proportion of participants aged 60 and older underwent specific balance testing. Study design and complete assessment procedures have been described in detail previously (19). Participants were ineligible for inclusion if they had one or more of the following conditions: a stroke within the last 12 months previous to the test date, focal lesions of the brain, delirium, inability to stand safely without support, considerable hearing problems, or any other restriction that would impair or prevent the completion of the testing such as dyslexia, uncorrected visual impairment, visual field impairment, neglect, or alcohol consumption before participation in the study.

All participants signed informed consent and received a small financial compensation. The study was in accordance with the declaration of Helsinki and received approval by the ethics committee of the University of Leipzig (approval numbers 263–2009-14122009, 263/09-ff, 201/17-ek).

### 2.2 Measures

#### 2.2.1 Exposure

Postural balance in humans is based on a dynamic coordinative interaction between sensory input from vestibular, visual and proprioceptive systems. The body corrects its posture based on this input. Static posturography objectively assesses postural balance by measuring the extent of body sways (changes in the center of pressure of the feet) under various test conditions, such as with open or closed eyes. The closed eye condition subtracts visual input. Therefore, the ratio of closed-eyed and open-eyed sway is an indicator of how much visual vs. vestibular and proprioceptive input contributes to the individual’s optimal static postural balance performance. Typically, the sway ratio is larger if the individual depends to a great extent on visual clues for postural stability, which may indicate the need to compensate for a lack of vestibular or proprioceptive input. Postural sway can be assessed using a platform that records the center of pressure and its shifts during the test paradigms while the tested individual stands unsupported on the platform (20). In the LIFE Adult Study postural control was assessed using a Zebris FDM-S power plate (zebris Medical GmbH, Isny im Allgäu, Germany) with a sampling rate of 50 Hertz analyzed by WinFDM-S. Sway path and sway area were direct outputs of the software. The Center of Pressure (COP) is calculated by the software from the combined load distribution under the feet. A 95% confidence ellipse is calculated from the time-varying points of the COP. As the COP is a time-varying point that moves as a person sways or adjusts, the ellipse captures the main area of this movement, indicating how much a person is shifting their weight and how stable they are. The sway path is the cumulative distance traveled by the COP as it moves over time. This path shows the trajectory of balance adjustments, with longer paths often indicating more movement or instability in balance. The participants were asked to stand on the force plate without shoes with their eyes open and their feet shoulder-width apart. This position was chosen because it corresponds to a natural posture and provides greater stability than a position in which both feet are close together or a fixed distance that is invariant to individual height. The position of the feet had to correspond to the illustration on the edge of the force plate. A one-minute measurement was carried out with documentation of the parameters area of the confidence ellipse in square millimeters (mm), and length of the sway path in mm. In an analogous manner, the participants were then asked to stand on the force plate with their eyes closed. The examiner had to ensure that the person was standing securely all the time. They remained in the immediate vicinity of the test person during the entire examination. The test was canceled if there was a risk of falling due to an unsteady stance. The examiner stood beside the participant to support them in the event of apparent instability, particularly in the closed-eye condition. There was no formal criterion for assessing postural instability other than the examiner’s judgment.

Romberg’s ratios were calculated by dividing closed-eyed sway path by open-eyed sway path and closed-eyed sway area by open-eyed sway area, respectively. These ratios can indicate a lack of vestibular and proprioceptive input to postural stability.

### 2.3 Outcome

To test executive, memory and processing speed functions, participants completed the CERAD-plus battery (Consortium to Establish a Registry for Alzheimer’s disease (21)). In accordance with previous studies we constructed summary scores for executive cognitive functions, memory performance and processing speed as follows: Executive function = [z phonemic fluency + z semantic fluency - z Trail Making Test (part B - part A)/part A]/3; memory performance = (z learning + z recall + z recognition)/3; processing speed = −z Trail Making Test part A, where z symbolizes the z score that was calculated to normalize raw scores to enable addition (22). Missing values were not replaced (23).

To test visuospatial function, a short form of the Judgment of Line Orientation Test (JLO, short form Q) was used (24). Participants are asked to match a pair of angled lines to the respective lines contained in a semi-circular fan of 11 lines. The short form Q scores add up to the number of correct items. This score is multiplied by two, one point is added in the 50–64 age group, three points are added in the 65–74 age group; two points are added in all age groups for females. Lower score values indicate higher impairment. JLO is a valid and reproducible test of isolated visuospatial abilities (25).

### 2.4 Covariates

#### 2.4.1 Age was defined at the first visit to the LIFE study center

An aggregated measure of socioeconomic status (SES) was operationalized from the dimensions education, income and occupation using a method established in large population-based surveys (26). This SES score ranges from 3.0 to 21.0 where lower values indicate lower SES. For the analysis, SES was categorized into low (SES ≤ 9.2), middle (9.2 < SES ≤15.5) and high (SES > 15.5). These thresholds correspond to quintiles where quintile 2 to 4 are aggregated, thus, the middle category comprises 60% of the population. SES was entered as a dummy variable into all multivariable analyses, with the lowest category serving as reference.

Comorbid diabetes was operationally defined as present (yes/no) if the patient reported having diabetes, or receiving antidiabetic medication, or fasting serum glucose was equal or above 7.0 mmoL/L, or HbA1c was equal or above 6.5%, or serum glucose was equal or above 11 mmoL/L measured 2 h after consuming 75 g of glucose solution (Accu-Chek® Dextrose O.G.T. Saft, Roche Diagnostics Deutschland).

APOE e4-allele carriers were identified by isolating genomic DNA from peripheral leukocytes, using an automated protocol on the Qiagen Autopure LS (Qiagen, Hilden, Germany). Genotyping of APOE allele status (e2, e3, e4) was performed using a Roche Lightcycler 480, following established methods (27).

Mild cognitive impairment (MCI) was ascertained using the International Working Group Criteria and expanded Mayo criteria (28, 29).

### 2.5 Statistical analysis

To examine the association between vestibular/proprioceptive balance control and neuropsychological test results, the Romberg’s ratios for sway path and sway area were tested in generalized linear regression models with the summary scores of, respectively, executive function, memory, and cognitive processing speed as dependent variables and in logistic regression models with the dichotomized score of spatial orientation (JLO) as dependent variable. As recommended (25) we dichotomized the JLO score using a score of 20 as cutoff value to minimize false positives. Visual inspection of the scatter plots of the bivariate associations was conducted to detect any apparent patterns of association such as deviations from the general straight-line assumption of linear regression.

Covariates for the models were chosen based on potential relevance for cognitive status or the association between cognition and postural control, namely sex, age, ApoE status, SES and history of stroke. As diabetes may impair postural stability by virtue of inducing polyneuropathy, we also included diabetes status into the models.

Significance level was set at two-sided alpha = 0.05. SAS V9.4 (SAS Institute Inc., Cary, NC, United States) was used for all analyses.

## 3 Results

In total, we analyzed 460 participants with a mean age of 68.6 years, range 60 to 80, 47.5% female. A total of 6.7% of participants had MCI. Sociodemographic characteristics are shown in Table 1. Cognitive test scores in detail are shown in Supplementary Table S1.

**Table 1:** Sociodemographic characteristics of participants, n = 460.

	*n*	
Female (%)	219	47.6
Age, mean (standard deviation/range)	460	68.6 (4.8/60.1–80.0)
Socioeconomic status (%)		
Low (SES < =9.2)	94	20.4
Middle (SES > 9,2 to 15,3)	276	60.0
High (SES > 15,3)	90	19.6
Anamnestic diabetes (%)	90	19.6
Anamnestic stroke (%)	17	3.7
ApoE* status (%)		
e2/e4	3	0.7
e3/e4	118	25.7
e4/e4	6	1.3

*Allele combinations of Apolipoprotein E.

Mean sway path was 219.3/519.9 mm for the open eyed/closed eyed condition, mean sway area was 42.0 mm^2^ for the open-eyed condition and 57.3 mm^2^ for the closed-eyed condition, resulting in a Romberg’s ratio of 2.4 for sway path and 1.6 for sway area (Table 2).

**Table 2:** Balance test characteristics of participants, mean (Standard deviation/range).

	*n*	Mean (SD/range)
Length of sway path (mm), eyes open	460	219.3 (101.4/59.9–875.0)
Length of sway path (mm), eyes closed	460	519.9 (320.4/102.1–2395.1)
Sway area, confidence ellipse (mm^2^), eyes open	460	42 (37.1/5.9–449.7)
Sway area, confidence ellipse (mm^2^), eyes closed	460	57.3 (55.1/7.6–832.3)
Romberg’s ratio, length of sway path	460	2.4 (1.2/0.6–13.1)
Romberg’s ratio, sway area, confidence ellipse	460	1.6 (1.1/0.1–7.5)

mm = millimeters.

A higher Romberg’s ratio for sway area and female sex were each significant and independent indicators for impaired visuospatial abilities as measured by the dichotomized Judgment of Line Orientation, JLO (Odds Ratio = 1.42 for sway area, Odds Ratio = 2.46 for female sex). Romberg’s ratio for sway path length was not significantly associated with JLO (see Table 3). Age, sociodemographic status, ApoE status, previous stroke and diabetes were not significantly associated with JLO. Sensitivity analyses using the JLO score as a continuous outcome yielded similar results.

**Table 3:** Association of two measures of vestibular function with visuospatial abilities (Judgment of Line Orientation, JLO), adjusted for potential confounders in two multiple logistic regression model.

	Visuospatial abilities	
	OR (95% CI)	OR (95% CI)
Measures of vestibular function		
Romberg’s ratio, sway area	**1.416 (1.067;1.881)**	
Romberg’s ratio, sway path		1.100 (0.793;1.526)
Covariates		
Female	**2.456 (1.082;5.577)**	2.166 (0.964;4.868)
Age (years)	1.005 (0.928;1.089)	1.003 (0.926;1.085)
Middle SES (SES > 9,2 -15,3) vs. low SES (SES < = 9,2)	0.565 (0.238;1.344)	0.581 (0.247;1.37)
High SES (SES > 15,3) vs. low SES (SES < = 9,2)	0.555 (0.171;1.803)	0.606 (0.19;1.933)
ApoE carrier	0.777 (0.318;1.895)	0.744 (0.307;1.803)
Anamnestic stroke	2.481 (0.516;11.933)	2.154 (0.449;10.323)
Diabetes*	0.812 (0.285;2.315)	0.841 (0.300;2.355)

SES, Socioeconomic status, OR, Odds Ratio; CI, confidence interval; ApoE, Apolipoprotein E. *Known diabetes or new diagnosis of diabetes as revealed by oral glucose tolerance/HBa1c (see Methods). JLO was dichotomized with a cut-off at 20 points. Higher Odds Ratios indicate a higher risk for impaired spatial orientation. Significant associations are printed in bold (*N* = 460).

Balance measures were not associated with executive, memory and processing speed functions. More advanced age was an independent significant indicator of worse memory and processing speed functions. Female sex was significantly associated with better memory; middle and high socioeconomic status were both associated with more favorable test results of executive functions and memory. Detailed results are shown in Table 4.

**Table 4:** Association of Romberg’s ratios with z-standardized a: executive function, b: memory function and c: procedural speed, adjusted for potential confounders in multiple linear regression models.

Executive functions *n* = 383						
	Estimate	SE	*p*- value	Estimate	SE	*p*- value
Measures of vestibular function						
Romberg’s ratio. Sway area	−0.0244	0.0309	0.4309			
Romberg’s ratio. Sway path				−0.0120	0.0296	0.6854
Covariates						
Female	0.0891	0.0716	0.2136	0.0929	0.0718	0.1957
Age (years)	0.0061	0.0075	0.4150	0.0063	0.0075	0.4019
Middle SES (SES > 9.2–15.3) vs. low SES (SES < = 9.2)	**0.2620**	**0.0886**	**0.0031**	**0.2613**	**0.0887**	**0.0032**
High SES (SES > 15.3) vs. low SES (SES < = 9.2)	**0.5603**	**0.1095**	**<0.0001**	**0.5563**	**0.1094**	**<0.0001**
ApoE carrier	0.0789	0.0750	0.2926	0.0820	0.0749	0.2739
Anamnestic stroke	0.1017	0.1979	0.6074	0.1106	0.1976	0.5757
Diabetes	−0.0702	0.0920	0.4451	−0.0733	0.0920	0.4253
Memory functions *n* = 385						
Measures of vestibular function	Estimate	SE	*p*- value	Estimate	SE	*p*- value
Romberg’s ratio. Sway area	−0.0411	0.0295	0.1637			
Romberg’s ratio. Sway path				0.0305	0.0281	0.2791
Covariates						
Female	**0.1643**	**0.0679**	**0.0156**	**0.1901**	**0.0681**	**0.0053**
Age (years)	**−0.0215**	**0.0071**	**0.0025**	**−0.0206**	**0.0071**	**0.0038**
Middle SES (SES > 9.2–15.3) vs. low SES (SES < = 9.2)	**0.3172**	**0.0837**	**0.0001**	**0.3211**	**0.0838**	**0.0001**
High SES (SES > 15.3) vs. low SES (SES < = 9.2)	**0.4484**	**0.1034**	**<0.0001**	**0.4368**	**0.1034**	**<0.0001**
ApoE carrier	−0.0465	0.0712	0.5137	−0.0420	0.0712	0.5556
Anamnestic stroke	−0.1681	0.1886	0.3727	−0.1345	0.1884	0.4753
Diabetes	−0.0086	0.0870	0.9210	−0.0112	0.0871	0.8980
Procedural speed *n* = 460						
Measures of vestibular function	Estimate	SE	*p*- value	Estimate	SE	*p*- value
Romberg’s ratio. Sway area	−0.0456	0.0380	0.2297			
Romberg’s ratio. Sway path				0.0275	0.0362	0.4476
Covariates						
Female	0.0285	0.0861	0.7405	0.0593	0.0866	0.4931
Age (years)	**−0.0481**	**0.0088**	**<0.0001**	**−0.0472**	**0.0088**	**<0.0001**
Middle SES (SES > 9.2–15.3) vs. low SES (SES < = 9.2)	0.1833	0.1051	0.0810	0.1864	0.1052	0.0764
High SES (SES > 15.3) vs. low SES (SES < = 9.2)	0.2289	0.1314	0.0815	0.2195	0.1315	0.0949
ApoE carrier	−0.0067	0.0921	0.9416	−0.0058	0.0922	0.9501
Anamnestic stroke	0.3090	0.2192	0.1585	0.3403	0.2196	0.1211
Diabetes*	**−0.2482**	**0.1060**	**0.0193**	**−0.2477**	**0.1062**	**0.0196**

SES, Socioeconomic status; SE, standard error. *Diabetes = Known diabetes or new diagnosis of diabetes as revealed by oral glucose tolerance/HBa1c (see Methods). Higher estimates indicate better cognitive function. Significant associations are printed in bold.

## 4 Discussion

The main result of this population-based study in adults aged 60 and older was that poorer postural balance during visual suppression, as indexed by larger sway area ratios during eyes closed relative to eyes open, was significantly and selectively associated with decreased visuospatial abilities as quantified by JLO, but not with functions in other cognitive domains such as memory or execution.

### 4.1 Non-visually guided postural balance and cognitive domains

In the LIFE Adult Study, postural balance was assessed with and without visual input. Sway parameters, specifically sway area, were calculated, which are sensitive even to subtle changes in postural stability (30). Postural imbalance upon visual suppression is indicative for a deficient vestibular or proprioceptive sensory feedback to central networks of postural control (31) Although dedicated vestibular testing was not performed, the following factors indirectly suggest that postural imbalance with eyes closed in the current study can be overly seen as a surrogate for peripheral vestibular deficits: (1) potential sources of proprioceptive impairment were corrected by adjusting for diabetes and age, (2) the Romberg’s ratio for sway area was associated with performance in the JLO. While the latter is considered a rather pure neuropsychological measure of visuospatial abilities, it has apparent similarities and overlaps with psychophysical testing of verticality perception based on the subjective visual vertical (SVV), which is considered a test of bilateral vestibular graviceptive input (32).

The specific association of postural balance without visual input and visuospatial abilities can be explained by taking a more detailed look at the pathways and networks underlying these functions. JLO engages the superior and posterior parts of the parietal cortex with a right-sided predominance (33). The same parietal areas are activated during mental rotation tasks and spatial updating by the precise estimation of distance and direction (34, 35). Furthermore, perception of verticality guided by graviceptive-vestibular inputs involves parieto-insular cortical areas (36). The superordinate and abstract function of these cortical areas is sensorimotor integration (33). This means that multisensory visual and vestibular afferent inputs are simultaneously processed, analyzed and hierarchically weighted with the major aim to achieve a global egocentric percept of the own body in environmental space. The latter is fundamental for balance control under different sensory conditions and further cognitive processes such as correct estimation of distances and directions in space with regard to the own position in space (37, 38). Thus, there seems to be an obvious link between multisensory, particularly higher-order vestibular processing, in the parietal cortex and visuospatial cognitive abilities. In line with this, vestibular function as tested by vestibular evoked myogenic potentials was predominantly associated with visuospatial abilities in a subcohort of the Baltimore Longitudinal Study of Aging (BLSA) (39).

In the current study, postural control during visual suppression did not show significant associations with executive functions, memory performance and processing speed, i.e., cognitive domains, which are commonly attributed to the frontal and temporal lobes. This finding seems to partially contradict previous reports on neuropsychological deficits in patients with unilateral and bilateral vestibulopathies, who showed mildly reduced performance in short-term memory, executive function, processing speed as well as visuospatial abilities (11) alongside postural imbalance during sensory perturbation. In a representative sample of 1,303 U.S. adults aged ≥60 years, vestibular dysfunction as measured by the modified Romberg’s test, similar to our study, was associated with worse performance in the digital symbol substitution (DSS) test (40). DSS is considered to test multiple cognitive domains including attention, visuospatial skills, associate learning and memory. The discrepancy of these findings to our results may be partially explained by the smaller sample size in the LIFE cohort. Furthermore, recent meta-analyses found heterogeneous associations of balance performance with multi-domain cognitive testing (10, 41). Arguably, more robust analyses are needed to allow a better understanding of the underlying mechanisms related to postural control. Also, the specific effects of the balance system need to be separated from more general effects of aerobic fitness and mobility on cognitive health.

### 4.2 Non-visually guided postural balance and cognitive decline

The predominant association of postural balance during visual suppression with neuropsychological deficits in the JLO may have interesting implications for the early diagnostic screening of cognitive decline and dementia. Previous studies show that in patients with Mild Cognitive Impairment (MCI), Alzheimer’s dementia (AD) and Parkinson’s Disease (PD) visuospatial abilities as quantified by JLO are inferior than in healthy controls and associated with a concomitant decrease in parietal gray matter volume (42, 43). In general, tests of visuospatial abilities such as the 4 Mountains Test for visual perception and mental rotation, the Rey-Osterrieth Complex Figure (ROCF test) for visuo-constructive abilities and complex visual memory as well as paradigms for spatial navigation, i.e., correct and efficient wayfinding and route execution, have been proven as highly sensitive screening tests for the early detection of MCI and also the differentiation of amyloid-positive and -negative MCI (44–46). Thus, postural imbalance as a marker associated with impaired spatial orientation could be an early sign of progressive dementia, thus, allowing early screening and intervention (47).

## 5 Strengths and limitations

In contrast to many of the previous studies, our analyses were carried out in a large, representative sample of a population-based study. This may make it less prone to bias from cohorts enriched in people with more comorbidities. The magnitude of the sway path and area found in our study lie within the expected range for an older, non-clinical population (48, 49). Other strengths of the study include the use of validated testing procedures along with deep phenotyping of participants. Also, participants with hearing impairment were excluded, confirming the postulated independent association between vestibular and cognitive functions (50), specifically regarding visuospatial abilities (51). One obvious limitation is the cross-sectional nature of the data which precludes the analysis of causal association and independent effects.

## 6 Conclusion

In our study of older adults, a population-based approach with a rigorous assessment routine aligns with previous studies investigating the association of vestibular with cognitive impairment conducted in clinical settings. We found that poorer postural balance during visual suppression was significantly and selectively associated with decreased visuospatial abilities. While a test for postural balance can only be viewed as a surrogate of vestibular function, it may be worthwhile to examine in the future whether inclusion of balance testing enhances the value of screening programs for early detection of cognitive impairment. Also, it may be appropriate to apply routine cognitive tests when balance problems are detected.

Balance functions and specifically vestibular functions are amenable to interventions. There is also evidence that cognitive training in MCI or early dementia helps to decelerate or even prevent cognitive deterioration (52, 53). Further research should investigate if balance is only an indicator, or if interventions targeted on balance are a necessary component to preserve brain health in aging.

## Data Availability

The raw data supporting the conclusions of this article will be made available by the authors, without undue reservation.
