# Supplementary material for: Postural imbalance without visual input is associated with specific neuropsychological deficits in older adults – results from the LIFE-adult study
Source: Front Neurol. 2024 Dec 11;15:1452150. doi: 10.3389/fneur.2024.1452150 (PMC11670201; doi:10.3389/fneur.2024.1452150)
Supplement: Supplementary file 1 [file Data_Sheet_1.pdf]

Supplementary Material

Supplementary Table S1. Raw values of cognitive test scores. Values of the Judgement of Line Orientation Test are presented corrected for age and gender.

	N	mean	SD	min	max
Semantic fluency (animals) (words)	459	22.7	5.9	7	42
Phonemic fluency (s-words) (words)	385	12.8	4.2	3	27
TMT part A (seconds)	459	41.8	14.4	17	133
TMT part B (seconds)	458	102.8	45.8	35	300
Word list learning (words)	385	22.2	3.2	14	30
Word list recall (words)	385	7.6	1.8	0	10
Word list recognition (words)	384	19.7	0.7	15	20
JLO (number of correct items)	460	27.7	4.4	11	35

TMT = Trail Making Test, JLO = Judgement of Line Orientation Test, short form Q, SD = Standard Deviation, min = minimum, max = maximum
